# Sotagliflozin attenuates cardiac dysfunction and remodeling in myocardial infarction rats

**DOI:** 10.1016/j.heliyon.2023.e22423

**Published:** 2023-11-15

**Authors:** Peng Zhong, Jingjing Zhang, Yanzhao Wei, Tao Liu, Minxiao Chen

**Affiliations:** a Department of Cardiology, Renmin Hospital of Wuhan University, Wuhan, 430060, China; b Department of Pharmacology, Renmin Hospital of Wuhan University, Wuhan, 430060, China

**Keywords:** Sotagliflozin, Myocardial infarction, Cardiac remodeling, Inflammation, SGLT1, SGLT2

## Abstract

**Objective:**

Sotagliflozin is a dual sodium-glucose co-transporter-1 and 2 (SGLT1/2) inhibitor with selectivity towards SGLT2. Previous studies showed that SGLT2 inhibitors can improve cardiac function and reduce myocardial infarction size in animal models of myocardial infarction (MI). However, it remains unknown whether the dual inhibition of SGLT1/2 by sotagliflozin has beneficial effects in this context. In this study, we investigated the potential cardioprotective effects of sotagliflozin in an animal model of MI.

**Methods:**

Sprague Dawley (SD) rats underwent left anterior descending coronary artery ligation or sham ligation then were randomly assigned to receive either sotagliflozin (10 mg/kg) or vehicle via intraperitoneal injection. Fourteen days post-MI, we assessed cardiac function using echocardiography and evaluated histological and molecular markers of cardiac remodeling and inflammation in the left ventricle.

**Results:**

Our findings indicate that sotagliflozin treatment resulted in improved cardiac function and reduced infarct size compared with the vehicle-treated group. Additionally, sotagliflozin improved cardiac remodeling as shown by the decreased cardiac hypertrophy and cardiac apoptosis in the post-MI heart. Mechanistically, an apparent reduction in the cardiac inflammatory response in sotagliflozin-treated hearts was observed in the post-MI rats.

**Conclusion:**

Overall, our results suggest that sotagliflozin may have cardioprotective effects against myocardial infarction.

## 1 Introduction

Sodium-glucose cotransporter 2 inhibitors (SGLT2i) are effective antidiabetic compounds that reduce heart failure hospitalization and cardiovascular death in patients with type 2 diabetes. While SGLT2i are designed to inhibit SGLT2 in the kidney, recent research has investigated their off-target cardiac effects. Although SGLT2 has not been found in the heart or cardiomyocytes, studies have shown that SGLT2i can still directly affect cardiomyocytes by reducing cytosolic [Ca^2+^] and [Na^+^], inhibiting NHE-1, and increasing mitochondrial function and cell viability. These effects contribute to the beneficial effects of SGLT2i under various pathological cardiac conditions [1].

Recent research indicates that SGLT1 is highly expressed in the hearts and cardiomyocytes of humans, rats, and mice [1]. Studies on murine models have shown that SGLT1 knockdown in cardiomyocytes can protect the heart from hypoxia/reoxygenation injury in vitro and ischemia-reperfusion injury in vivo and ex vivo [2]. This effect is attributed to decreased oxidative stress, myocardial necrosis, infarction size, and improved hemodynamic function [2]. In contrast, cardiac overexpression of SGLT1 in mice increased markers of cardiac hypertrophy, glycogen content, and heart ratio, resulting in progressive left ventricular dysfunction, myocyte enlargement, and interstitial fibrosis [3]. Collectively, these findings suggest that SGLT1 inhibition may have cardiac benefits.

However, a recent study showed pharmacological dual SGLT1/2 inhibition with T-1095 worsened cardiac dysfunction following myocardial infarction in a murine model [4]. T-1095 is a prodrug that can be readily absorbed from the small intestine before conversion to its active form, T-1095A, systemically. T-1095A nearly equally inhibits SGLT1 and SGLT2, with only a 4-fold selectivity for SGLT2 versus SGLT1, unlike the widely used SGLT2 inhibitors canagliflozin, dapagliflozin and empagliflozin which have far higher SGLT2 vs SGLT1 selectivity ratios exceeding 250-, 1200-, and 2500-fold, respectively [5,6]. This study raised concerns regarding the effects of dual SGLT1/2 inhibitors in myocardial infarction conditions.

Sotagliflozin is a dual sodium-glucose co-transporter-2 and 1 (SGLT2/1) inhibitor with 20-fold higher selectivity for SGLT2compared to SGLT1 [7]. Clinical studies have shown beneficial cardiorenal effects of sotagliflozin in type 2 diabetes [8].Additionally, compared to pure SGLT2 inhibitors, dual SGLT1/2 inhibitors demonstrated lower myocardial infarction risk in diabetic patients [9]. A recent animal study showed sotagliflozin could reduce cardiac fibrosis and hypertrophy evoked by pressure overload in a normoglycemic mouse model, suggesting that the cardioprotective effects of dual SGLT1/2 inhibition could also extend to nondiabetic patients [10]. As SGLT2 inhibitors have been demonstrated to reduce myocardial infarction size and cardiac remodeling, coupled with improved systolic function in both diabetic and non-diabetic animal models of myocardial infarction [11], whether dual SGLT1/2 inhibition by sotagliflozin has any beneficial effects is still unknown. Based on previous negative results of T-1095 on myocardial infarction conditions, it is important to further determine the effects of sotagliflozin in this scenario. In this study, we aimed to determine the potential effects of sotagliflozin on the heart in an animal model of myocardial infarction.

## 2 Methods

### Ethical approval

Male Sprague-Dawley (SD) rats (180–220 g) purchased from the HFK Biotechnology Company (Beijing, China) were used. All animal experiments were approved by the Animal Care and Use Committee of Renmin Hospital of Wuhan University and were conducted under the National Institutes of Health (NIH) Guide for the Care and Use of Laboratory Animals (NIH Publications No.8023, revised 1978). Rats were housed under conditions of 22 ± 2.0 °C, 50 % ± 5 % humidity and a 12-h light/12-h dark cycle, with ad libitum access to food and water. At experiment end, the rats were anesthetized by deep anesthesia with 4 % isoflurane in a bell jar before heart removal.

### 2.1 Myocardial infarction (MI) model

Male 6-week-old SD rats were anesthetized with pentobarbital sodium (40 mg/kg, i.p.) and intubated for ventilation using a Zoovent ventilator. Rats were randomly divided into three groups: sham, MI + saline, and MI + sortagliflozin. Myocardial infarction (MI) was induced via proximal left anterior descending coronary artery (LAD) ligation as described by Yang et al. [8]. Electrocardiogram ST-segment elevation confirmed successful artery occlusion. The MI + sotagliflozin group received sotagliflozin (10 mg/kg/day) intraperitoneal for 2 weeks immediately after MI surgery. The sham group underwent identical procedures without LAD ligation as sham-operated controls.

### 2.2 Echocardiography

Echocardiography was conducted under anesthesia using a 30-MHz probe with a Vevo2100 imaging system (Visual Sonics Inc., Toronto, ON, Canada). Prior to transthoracic echocardiography performed by an experienced technician, rats were anesthetized with 40 mg/kg pentobarbital sodium administered intraperitoneally. Left ventricular ejection fraction (LVEF) and left ventricular internal diameter end-diastole (LVIDd) were calculated using the echocardiographic system.

### 2.3 Histological analysis

At experiment end, rats were euthanized and the hearts arrested in diastole by hyperkalemic solution (19 % KCl) infusion followed by 24h buffered formalin immersion. Median papillary muscles slices were dehydrated, paraffin-embedded, and 5-μm serially sectioned. Sirius red staining evaluated collagen deposition. CD68 antibody staining followed by FITC-conjugated secondary antibody assessed macrophage infiltration. Nuclei were DAPI counterstained and sections fluorescently examined. Infarction size was measured as the percentage of LV circumference from Sirius red-stained sections 14 days post-MI. TUNEL assay detected DNA fragmentation using the Apoptag kit per manufacturers protocol.

### 2.4 Western blot analysis

Total protein from the Left ventricular was extracted and its concentration measured using the Pierce BCA Protein Assay Kit (Pierce). Fifty micrograms of the extracted protein was subjected to SDS-PAGE (Invitrogen) and transfer onto a polyvinylidene fluoride membrane (Millipore), followed by overnight incubation at 4 °C with an array of primary antibodies [IL-1β (ab9722), obtained from Abcam; Bax (sc-7480), Bcl2(sc-23959), ANP (sc-515,701), BNP (sc-271,185, p-P65 (sc-8008), and GAPDH (sc-47724) obtained from Santa Cruz; cleaved-caspase 3 (#9661), NLRP3 (#15101), IL-18 (#67775), TNF-⍺ (#3707) from Cell Signaling]. Membranes were then incubated 2h at room temperature with HRP-conjugated secondary antibodies and visualized using ECL reagent (Bio-Rad). GAPDH was used as a loading control for protein normalization.

### 2.5 ELISA

ELISA was used to determine N-terminal prohormone of brain natriuretic peptide (NT-proBNP) (#CEA485Ra, Cloud-Clone) expression in rat plasma. Briefly, samples were collected in anticoagulant-containing tubes, centrifuged, and supernatants collected. 100 μl supernatant was added to wells for incubation, followed by washing and sequential addition of detection antibody. Tetramethylbenzidine (TMB) substrate was then used for colorimetric detection. Absorbance was measured by spectrophotometer at 450 nm.

### 2.6 Statistical analysis

Statistical significance was calculated via one-way ANOVA with Tukey's Multiple Comparison Test.

Data are represented as mean ± SEM. Statistical analyses were performed using GraphPad Pro5.0. All p value of <0.05 were considered significant.

## 3 Results

### 3.1 Sotagliflozin treatment attenuated cardiac dysfunction in post-MI rats

**Fig. 1:**
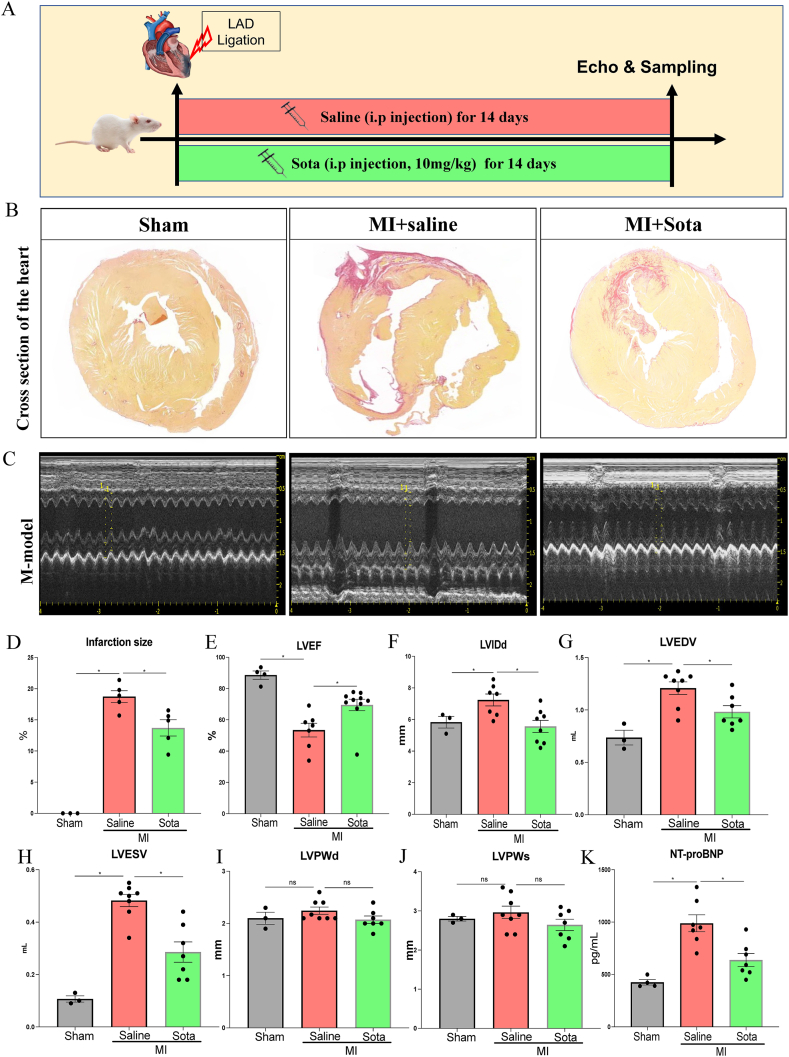
**Effects of sotagliflozin on cardiac function in post-MI rats.** (A) Diagram of experimental design. (B) Representative Sirius red-stained images of the heart at 14 days post-MI. (C) Representative M-mode echocardiogram obtained at the end of the experiment. (D) Quantitative infarction size based on Sirius red-stained sections between different groups. (E) Left ventricular ejection fraction (LVEF). (F) Left ventricular internal diameter at end diastole (LVIDd). (G) Left ventricular end-diastolic volume (LVEDV). (H) Left ventricular end-systolic volume (LVESV). (I) Left ventricular posterior wall thickness at end diastole (LVPWd). (J) Left ventricular posterior wall thickness at end systole (LVPWs). (K) Serum NT-proBNP level. Data are expressed as the mean ± SEM; n = 3–8 in each group. *p < 0.5; Sota: sotagliflozin.

In examining the gross morphology of hearts harvested from the different groups, we found that the ligation of LAD successfully induced infarction (as evidenced by the scar area) in both the MI + saline and MI + sotagliflozin groups (Fig. 1A). However, the degree of infarction size (quantification of the scar area) in rats from the MI + saline group was significantly higher than that in rats from the MI + sotagliflozin group (Fig. 1B and D). Echocardiography analysis conducted two weeks post-myocardial infarction demonstrated that rats in the MI group exhibited significantly diminished left ventricular function and cavitary dilation compared to sham-operated controls, as evidenced by decrements in LVEF, coupled with augmentations in LVIDd, LVEDV, and LVESV in MI rats relative to shams (Fig. 1B–H). Interestingly, treatment with sotagliflozin significantly improved cardiac dysfunction and dilation compared with that in the saline-treated group (Fig. 1B–H). No intergroup differences were observed in left ventricular posterior wall thickness (Fig. 1I and J). In accordance with the echocardiography findings pertaining to cardiac function, the MI + saline group exhibited a notable increase in the serum NT-proBNP level, which, however, demonstrated significant improvement after sotagliflozin treatment (Fig. 1K). Taken together, these results indicate that treatment with sotagliflozin attenuates cardiac dysfunction in post-MI rats.

### 3.2 Sotagliflozin treatment reduced cardiac hypertrophy and apoptosis in post-MI rats

**Fig. 2:**
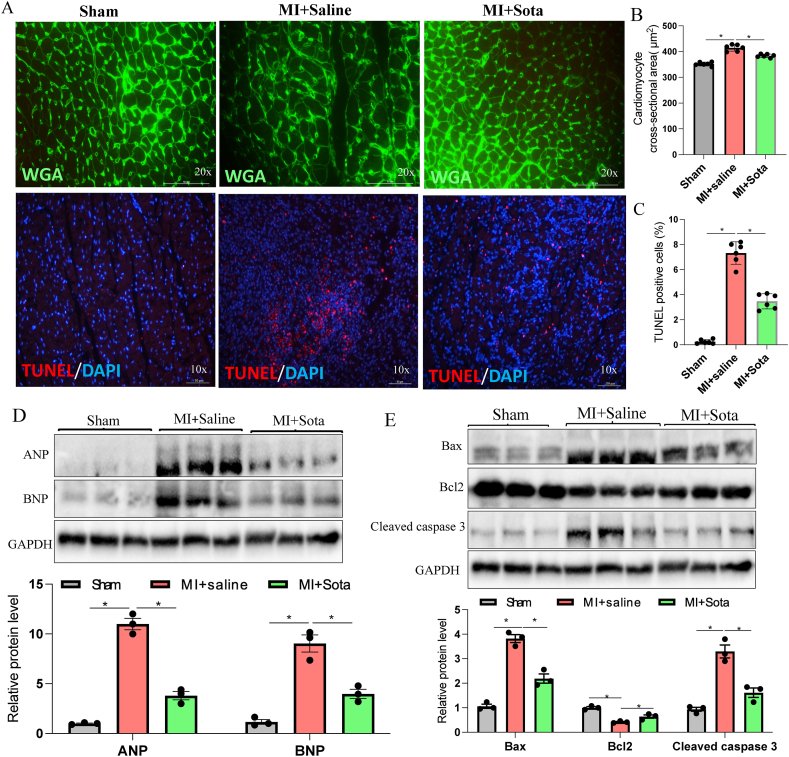
**Effects of sotagliflozin on cardiac hypertrophy and apoptosis in post-MI rats.** (A) Representative images of wheat germ agglutinin (WGA) and TUNEL staining of the heart. (B) Quantification of the cardiomyocyte cross-sectional area. (C) Quantitative analysis of TUNEL-positive cells in the heart. (D) Western blot analysis of hypertrophic markers (ANP and BNP) in the heart. (E) Western blot analysis of apoptosis markers (Bax, Bcl2, and Cleaved caspase 3) in the heart. Data are expressed as the mean ± SEM; n ≥ 3 in each group. *p < 0.5; Sota: sotagliflozin.

Cardiac hypertrophy and apoptosis are important in post-MI cardiac remodeling pathogenesis. Histological cardiomyocyte cross-sectional area analysis by WGA staining showed increased cell size in MI + saline versus sham group, indicating hypertrophy (Fig. 2A and B). Furthermore, ANP and BNP hypertrophic gene expression was upregulated in saline-treated MI hearts compared to sham (Fig. 2D). However, cardiomyocyte size and hypertrophic gene expression were significantly reduced in sotagliflozin-treated post-MI hearts (Fig. 2A–D). Additionally, more TUNEL-positive cells were found in non-infarcted areas in MI + saline group versus sham, while sotagliflozin dramatically reduced the number of TUNEL-positive cells in the post-MI heart (Fig. 2A and C). Moreover, apoptosis-related proteins (Bax, Bcl2, and cleaved caspase 3) were evaluated in the peri-infarction region. As shown in Fig. 2E, pro-apoptotic markers cleaved caspase 3 and Bax were significantly elevated in the MI + saline group, coupled with downregulation of the anti-apoptotic marker Bcl2. Interestingly, all of these changes were resolved by sotagliflozin treatment. Collectively, these results indicate sotagliflozin prevents cardiac hypertrophy and apoptosis in the post-MI hearts.

### 3.3 Sotagliflozin treatment attenuated cardiac inflammation in post-MI rats

**Fig. 3:**
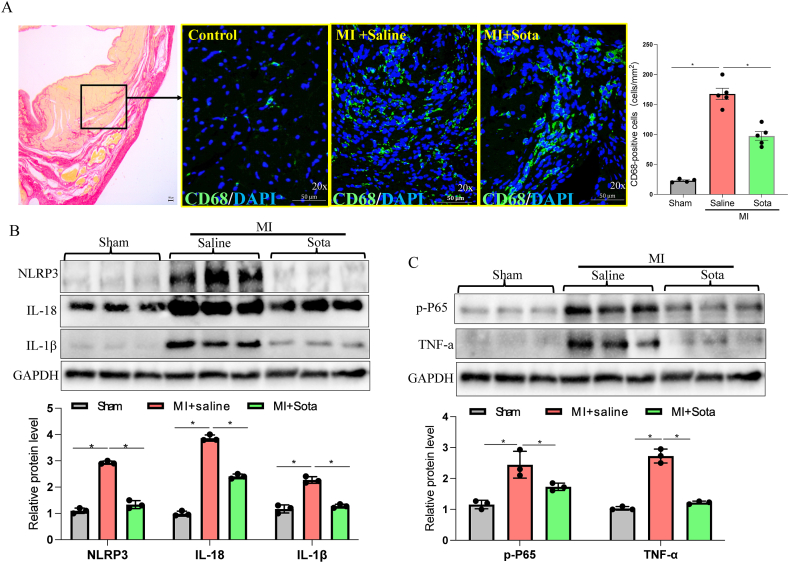
**Effects of sotagliflozin on cardiac inflammation in post-MI rats.** (A) Representative images of heart tissue stained with Sirius red or CD68 fluorescent antibody (green) and DAPI (blue) from post-MI rats treated with sotagliflozin or saline. (B) Western blot analysis of the NLRP3 pathway markers (NLRP3, IL-18, and IL-1β) in the peri-infarction region of the heart. (C) Western blot analysis of NF-κB pathway markers (p-P65, TNF-α) in the peri-infarction region of the heart. Data are expressed as the mean ± SEM; n ≥ 3 in each group. *p < 0.5. Sota: sotagliflozin.

The inflammatory response to acute MI determines MI size, and persistent inflammation can contribute to adverse post-MI remodeling [12]. We then evaluated the inflammatory response in the peri-infarction region of the post-MI rats. As shown in Fig. 3A, immunofluorescence staining of CD68, a marker of macrophages, showed a large amount of CD68^+^ macrophage infiltration in the peri-infarction region in the MI group, while this phenomenon was attenuated in the sotagliflozin-treated group (Fig. 3A). In addition, the protein levels of inflammatory factors in the heart tissues were determined. Interestingly, the protein levels of NLRP3, IL-18, IL-1β, p-P65 and TNF-⍺ were all significantly increased in the MI + saline group but were strikingly suppressed by sotagliflozin treatment (Fig. 3B–C). Collectively, these results indicated an anti-inflammatory effect of sotagliflozin in the context of the MI condition.

## 4 Discussion

In this study, our findings showed that treatment with sotagliflozin showed beneficial effects in the post-MI condition, as evidenced by the improved cardiac function and reduced infarction size in the post-MI rats compared with the vehicle-treated group. In addition, sotagliflozin improved cardiac remodeling, as shown by the decreased cardiac hypertrophy and cardiac apoptosis in the post-MI heart. Mechanistically, we observed an apparent reduction in the cardiac inflammatory response in the sotagliflozin-treated hearts of post-MI rats. Collectively, our results demonstrated a protective effect of sotagliflozin on the heart under MI conditions.

Under physiological conditions, SGLT1 is Chiefly expressed on the apical brush border membrane of enterocytes lining the small intestine. It co-transports one d-glucose or galactose molecule along with two sodium ions from the intestinal lumen into mature enterocytes of the small intestine. This translocation is responsible for absorbing dietary glucose and galactose. Meanwhile, SGLT2 resides predominantly in the luminal membrane of the S1 and S2 renal proximal convoluted tubule segments. It co-transports single filtered d-glucose molecules with single sodium ions into proximal tubule epithelial cells. This reabsorbs ∼90 % of filtered glucose. The remaining ∼10 % is subsequently absorbed by SGLT1 in the S3 segment, where luminal glucose content are reduced [13]. Based on the pivotal role of SGLT in glucose regulation, pharmacological targeting of SGLT has been pursued. Phlorizin, the first naturally discovered O-glucoside inhibitor of SGLT1/2, displays approximately 13 times more selectivity for SGLT2 over SGLT1. Studies have demonstrated that phlorizin can improve insulin resistance and diabetic complications. However, Phlorizin has several limitations, including poor intestinal absorption, low oral bioavailability, rapid urinary clearance, instability, and poor water solubility, precluding its clinical application as an anti-diabetic medication. Since SGLT1 mutations cause impaired intestinal glucose-galactose assimilation and lethal diarrhea, while SGLT2 mutations do not appear harmful, research has focused on developing other glycoside-based molecules with greater SGLT2 affinity that could also overcome phlorizin's pharmacokinetic shortcomings [14]. T-1095, with approximately 4-fold higher selectivity for SGLT2 over SGLT1, was the first orally absorbable O-glucoside SGLT2 inhibitor developed to surmount phlorizin's drawbacks. However, O-glucosides remain unstable metabolically because they can be cleaved by β-glucosidases. In contrast, C-glucosides have greater metabolic stability, improved oral bioavailability, and comparable SGLT2 inhibitory potency, enabling lower dosing for equivalent effects. Thus, after phase II trials, T-1095 was discontinued and superseded by a new generation of C-glucosides. Currently, all SGLT2 inhibitors approved for treating T2DM are all C-glucosides such as dapagliflozin (approximately 1200-fold selectivity for SGLT2 versus SGLT1), canagliflozin (∼155-fold selectivity for SGLT2 over SGLT1), empagliflozin (∼2600-fold selectivity for SGLT2 over SGLT1), and sotagliflozin (∼20-fold selectivity for SGLT2 over SGLT1).

SGLT2 inhibitors are now recommended foundational therapy for heart failure with reduced ejection fraction (HFrEF) due to favorable mortality, clinical events, and quality of life impacts [15]. Guidelines endorse dapagliflozin/empagliflozin for all HFrEF patients or sotagliflozin for those with diabetes [15]. SGLT2 inhibitors are generally safe, with minimal blood pressure effects, glycemia adverse events, and no acute kidney injury excess in trials. The rapid event curve separation in trials (2–3 months) indicates a glucose-lowering independent mechanism [16]. A recent study showed that the SGLT2 inhibitor empagliflozin can improve heart failure and adverse cardiac remodeling by enhancing the energy status of the myocardium by shifting the heart's metabolic substrates from glucose to fatty acids, ketone bodies, and branched-chain amino acids in a non-diabetic porcine model of myocardial infarction [17]. These results suggest direct cardioprotective effects of SGLT2i on the heart under MI conditions.

Unlike SGLT2, which is restricted to the kidneys, SGLT1 is present at lower levels in the human heart, primarily in the sarcolemma [18]. Insulin can promote SGLT1 translocation to the sarcolemma by activating protein kinase C and phosphorylating SGLT1, thus increasing glucose uptake. Recent studies indicate SGLT1 plays a key role in ischemic heart disease. SGLT1 expression increases 2- to 3-fold in myocardial ischemia [19]. Notably, SGLT1 knockdown decreased oxidative stress, necrosis, and infarct size after ischemia-reperfusion injury [2]. During ischemia, AMPK upregulates SGLT1 via ERK. SGLT1 then interacts with EGFP, elevating PKC and Nox2 activity and oxidative stress. Additionally, other disease models support the harmful effects of SGLT1. Adverse cardiac remodeling from transverse aortic constriction is mitigated in SGLT1 knockout mice [20]. Knockout of SGLT1 also attenuates cardiac hypertrophy in neonatal mouse hearts stimulated by an adrenergic ⍺1 receptor agonist [20]. In contrast, chronic SGLT1 overexpression causes glycogen storage cardiomyopathy resulting in pathological hypertrophy and impaired cardiac function. Pharmacological and genetic SGLT1 inhibition prevents this harmful cardiomyopathy phenotype [3,21]. Collectively, these findings indicate SGLT1 exerts pro-hypertrophic and detrimental effects on cardiomyocytes under conditions of cellular stress. Thus, the attenuated hypertrophic effects observed in diabetic cardiac tissue may be attributable, at least in part, to sotagliflozin-mediated inhibition of SGLT1 activity.

As shown in the present study, a cardioprotective effect of sotagliflozin was observed in the MI condition, as evidenced by the reduced cardiac dysfunction and pathological remodeling, suggesting that sotagliflozin may have therapeutic potential in ischemic heart diseases. In addition, the two proinflammatory pathways, NLRP3 inflammasome and NF-κB/TNF-⍺ signaling cascade were significantly inhibited by sotagliflozin treatment in the post-MI heart. The NLRP3 inflammasome is an innate immune multiprotein complex chiefly triggered by pathogen infection and cellular damage. It composes of the innate immune receptor protein NLRP3, adaptor protein ASC, and inflammatory protease caspase-1. The assembled NLRP3 inflammasome can activate the protease-1 to induce gasdermin D-dependent pyroptosis and facilitate the release of IL-1β and IL-18, the end products of NLRP3 activation, provoking a cytokine storm. NLRP3 activation in acute MI can promote adverse cardiac remodeling, systolic dysfunction, and heart failure [22]. Furthermore, the NF-κB/TNF-⍺ pathway has been shown to play a key part in myocardial ischemia/reperfusion injury and pathological post-infarction cardiac remodeling [23,24]. Overall, these findings indicate an anti-inflammatory effect for sotagliflozin, congruent with the established anti-inflammatory properties of other SGLT2 inhibitors like dapagliflozin, empagliflozin, and canagliflozin in vitro and in vivo [13].

In summary, our study demonstrated the therapeutic potential of sotagliflozin in MI conditions, coupled with an anti-inflammatory role in the heart. These results support further utilization of sotagliflozin in human ischemic diseases.

## Ethics approval and consent to participate

The present study was conducted according to the “Guide for the Care and Use of Laboratory Animals” (NIH Publication No.85–23, revised in 2010). The animal experiments were approved by the Laboratory Animal Welfare and Ethics Committee of Renmin Hospital of 10.13039/501100007046Wuhan University. Authors give full approval and consent to participate.

## Consent for publication

Authors give full consent for publication.

## Availability of data and materials

The datasets used and analyzed during the current study are available from the corresponding author on reasonable request.

## Funding

This work was supported by financial support from 10.13039/501100007046Wuhan University [Grant number: 2042019kf0074].

## CRediT authorship contribution statement

**Peng Zhong:** Conceptualization, Funding acquisition, Supervision, Writing – original draft. **Jingjing Zhang:** Data curation, Investigation, Methodology, Visualization. **Yanzhao Wei:** Investigation. **Tao Liu:** Writing – review & editing. **Minxiao Chen:** Formal analysis, Investigation.

## Declaration of competing interest

The authors declare the following financial interests/personal relationships which may be considered as potential competing interests:Peng Zhong reports financial support was provided by 10.13039/501100007046Wuhan University; 10.13039/501100016345Renmin Hospital.
