# Supplementary figures and images for: Sotagliflozin attenuates cardiac dysfunction and remodeling in myocardial infarction rats
Source: Heliyon. 2023 Nov 15;9(11):e22423. doi: 10.1016/j.heliyon.2023.e22423 (PMC10696107; doi:10.1016/j.heliyon.2023.e22423)

ANP

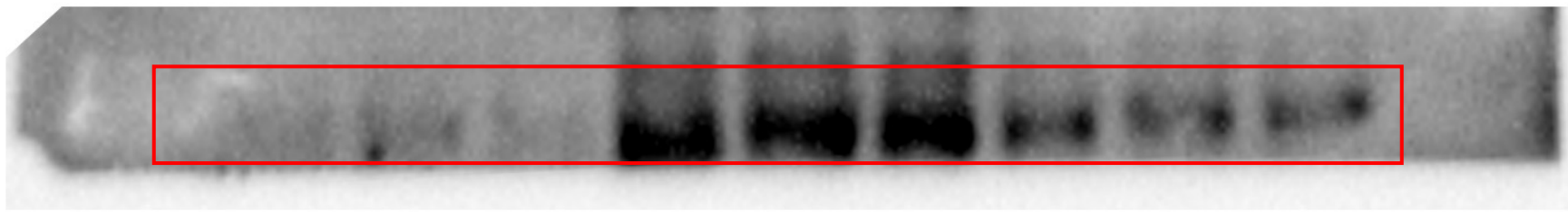

BNP

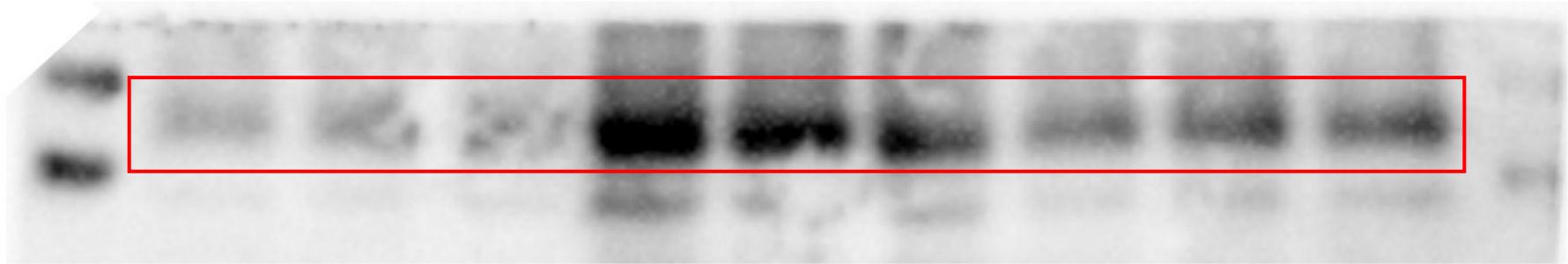

GAPDH

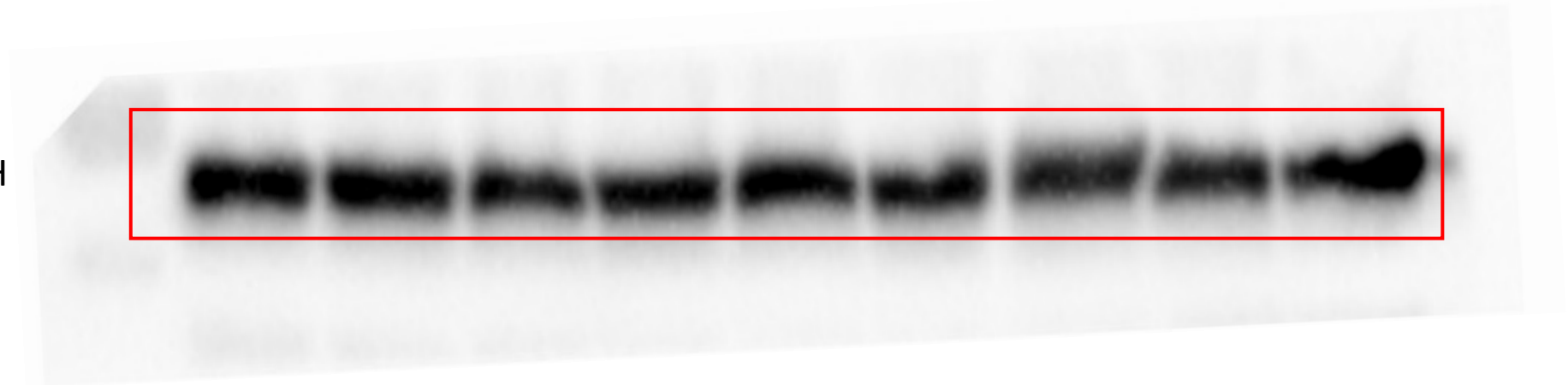

Bax

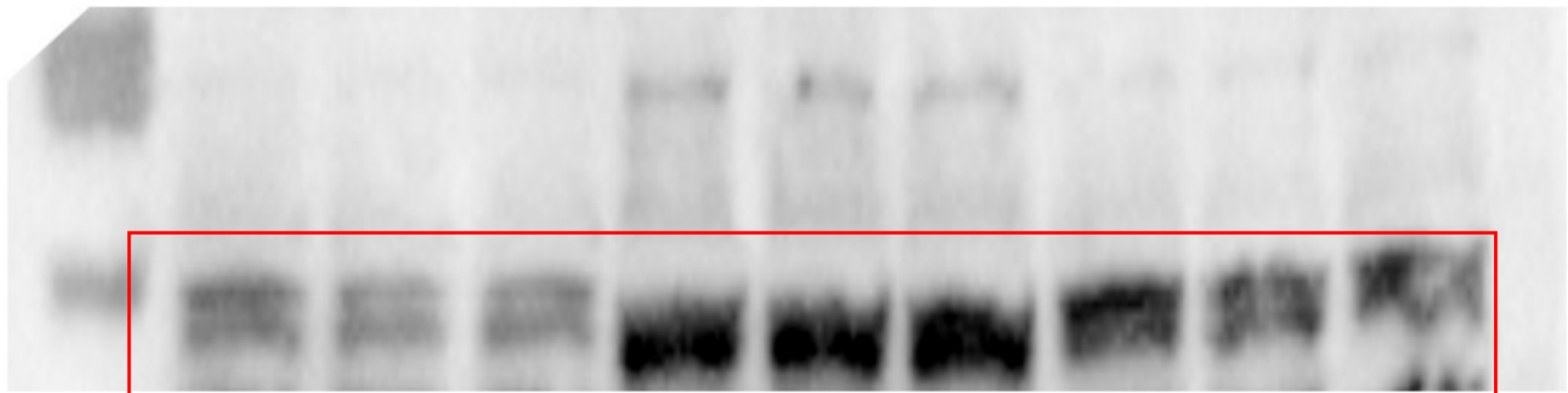

BCI2

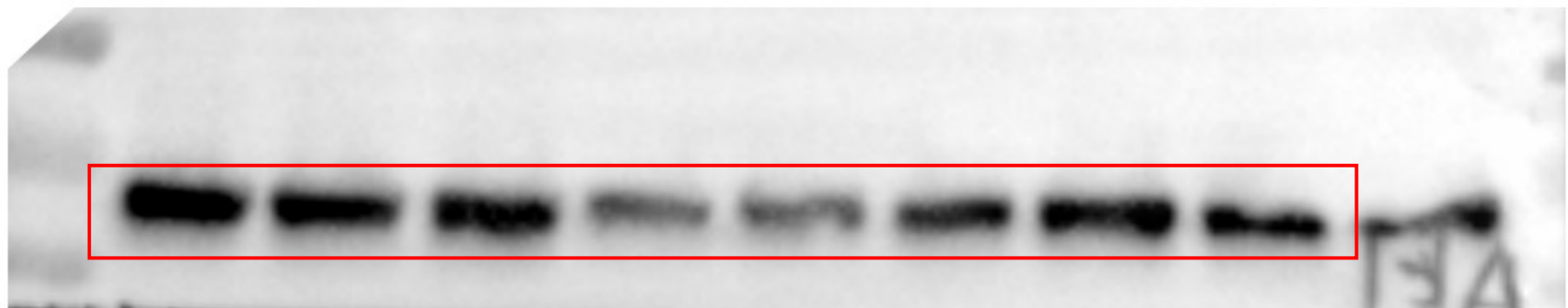

Cleaved caspase 3

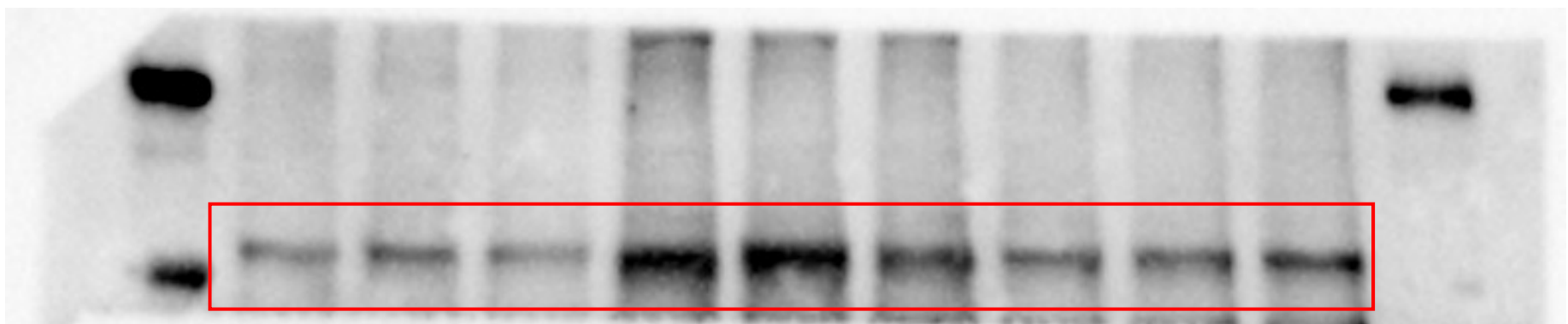

NLRP3

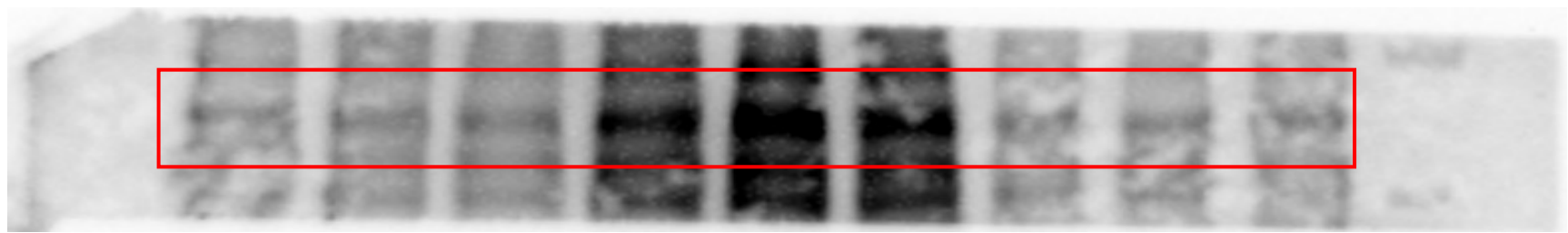

IL-18

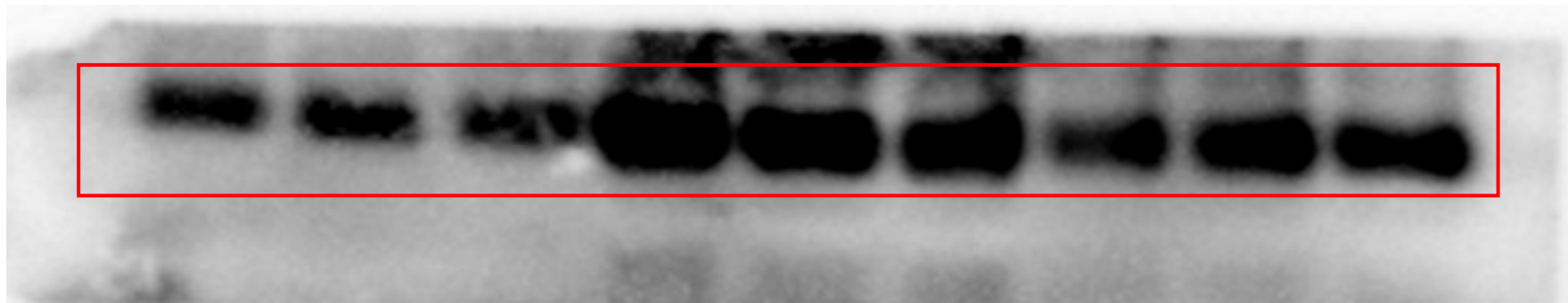

IL-1b

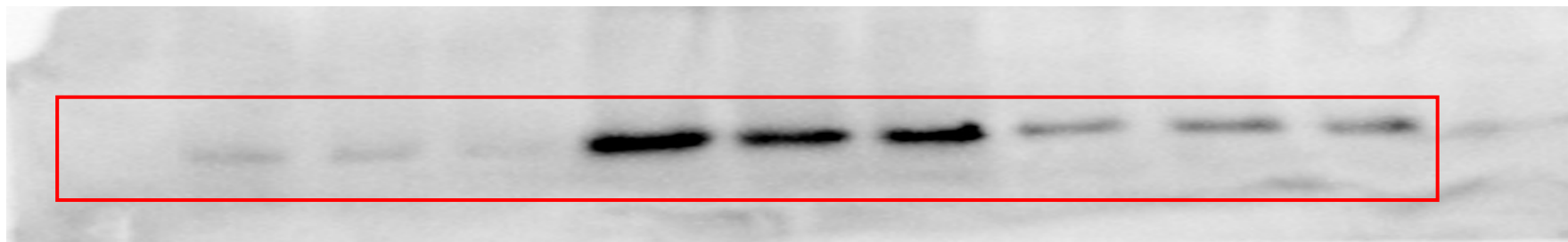

P65

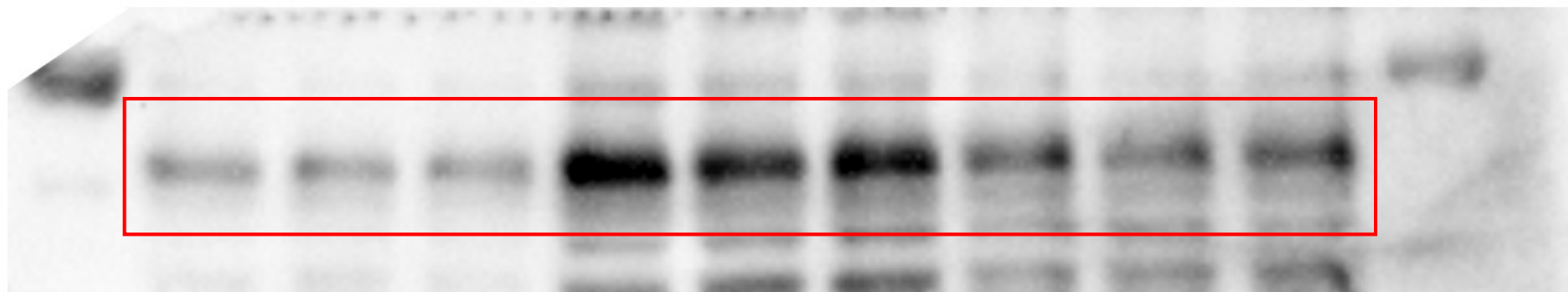

TNF- $\alpha$

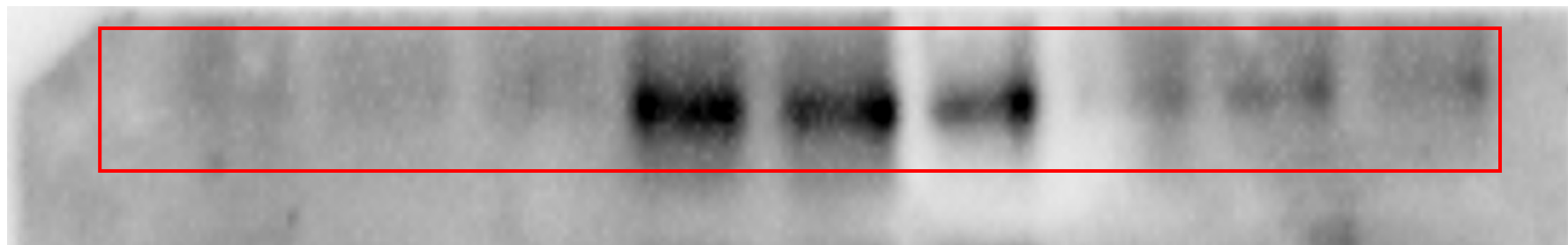

Supplement: Multimedia component 1 [file mmc1.pdf]
